# Translation, Adaptation, and Determining the Intra-Rater Reliability of the Balance Evaluation Systems Test (BESTest) for Persian Patients with Chronic Stroke

**DOI:** 10.3390/brainsci13121674

**Published:** 2023-12-04

**Authors:** Mansoureh Sadat Dadbakhsh, Afarin Haghparast, Noureddin Nakhostin Ansari, Amin Nakhostin-Ansari, Soofia Naghdi

**Affiliations:** 1 Department of Physiotherapy, School of Rehabilitation, Tehran University of Medical Sciences, Tehran 1417613151, Iran; m-dadbakhsh@razi.tums.ac.ir; 2 Sports Medicine Research Center, Neuroscience Institute, Tehran University of Medical Sciences, Tehran 1417613151, Iran; haghparast.afarin@gmail.com (A.H.); a-nansari@alumnus.tums.ac.ir (A.N.-A.); 3 Research Center for War-Affected People, Tehran University of Medical Sciences, Tehran 1417613151, Iran; nakhostin@tums.ac.ir

**Keywords:** BESTest, stroke, Persian, reliability

## Abstract

This study aimed to translate and culturally adapt the BESTest to the Persian language and evaluate its intra-rater reliability in Iranian patients with stroke. A forward-backward translation and expert panel review method was followed. Eighteen patients post-stroke (15 men, 3 female) were included which were assessed by a physiotherapist two times with a one-week interval. The mean total score for the test and retest were 83.66 (SD = 11.98) and 82 (SD = 13.23), respectively. There were no floor and ceiling effects. The intra-rater ICC for the total score was 0.88 (95% CI = 0.73–0.95). The ICC for the BESTest sections ranged from 0.55 (95% CI = 0.12–0.80) to 0.89 (95% CI = 0.55–0.96). The standard error of measurement and the smallest detectable change of the BESTest total score were 8.33 and 22.82, respectively. Our findings confirm the intra-rater reliability of the Persian BESTest for balance assessment of patients with chronic stroke.

## 1. Introduction

A stroke is an acute impairment of brain function due to a disruption in blood supply to the brain [1]. In 2017, the incidence of stroke was estimated to be about 11.9 million, and it was the second cause of death in 2019 [2,3]. The trend in the prevalence of stroke in the Middle East and North Africa (MENA) region closely mirrors the global pattern, with a gradual decrease in recent years [4]. Nevertheless, the majority of the stroke burden is concentrated in lower-income and lower-middle-income countries [3]. In Iran, stroke stands out as a significant contributor to disabilities, and its annual incidence varies widely across different regions of the country, spanning from 23 to 103 cases per 100,000 people [5,6]. Hence, stroke stands as a significant contributor to both disability and mortality on a global scale and within Iran.

Stroke could result in life-long persistent physical, psychological, and cognitive impairments [7], with motor disorders affecting approximately 80% to 90% of stroke survivors, making it a significant cause of disability [8]. Among these physical impairments, balance issues are among the most prevalent, affecting an estimated 87.5% of stroke patients [8]. These balance problems are often attributed to weakened muscles, abnormal muscle tone, sensory deficits, cognitive issues, and delayed automatic postural responses [9]. Notably, post-stroke balance problems elevate the risk of falls [10], further impacting the quality of life and increasing healthcare expenses [11,12]. Hence, the assessment of balance in patients with stroke using reliable and valid tools are crucial to monitor changes after using rehabilitation interventions [13,14,15].

Using a reliable and valid assessment tool for balance is crucial in post-stroke survivors, as balance impairment significantly increases the risk of falls in these individuals [9]. Such assessment tools can help identify the balance issues that would benefit from rehabilitation, determine the specific system contributing to the balance impairment, and monitor progress during treatment [16,17,18,19]. Several balance assessment tools have been employed in stroke patients, including the Berg Balance Scale (BBS), Postural Assessment Scale for Stroke Patients (PASS), Community Balance and Mobility Scale (CB & M), Timed Up and Go (TUG), and Dynamic Gait Index (DGI). However, each of these tests has its limitations [20,21]. The BBS is considered the gold standard for balance assessment, but it does not encompass dynamic balance [22,23]. PASS and CB & M exhibit ceiling and floor effects, respectively [23]. TUG serves as a screening test but lacks an in-depth evaluation of the balance system [24]. The complexity of the balance system makes it challenging to determine the specific system responsible for balance impairment. An important drawback of the current balance tests is their inability to evaluate the particular systems contributing to balance impairments [25].

Balance Evaluation Systems Test (BESTest) is a test developed by Horak et al. to identify the disordered systems responsible for poor balance control. The BESTest has 36 items designed to evaluate the performance of six balance systems of biomechanical constraints, stability limits/verticality, transition/anticipatory postural adjustment, reactive postural responses, sensory orientation, and stability during gait [21,25].

Post-stroke survivors experience a range of issues, which can more or less affect all the systems evaluated in the BESTest [26,27,28]. Previous studies have demonstrated the BESTest has no floor or ceiling effects [29] and is reliable, valid, and responsive to change for assessing balance in patients with stroke [21,25]. BESTest has been translated and culturally adapted into various languages, including German [29], Spanish [30], Norwegian [31], Spanish [32] and Korean [33]. There is no prior research on translating and culturally adapting the BESTest into the Persian language. Therefore, the aim of this study was to translate the BESTest into Persian and assess its intra-rater reliability among patients with stroke.

## 2. Materials & Methods

### 2.1. Design

A cross-sectional study was carried out to develop the Persian version of the BESTest and to examine its intra-rater reliability (i.e., between-day reliability) in patients with stroke. The study protocol was approved by the Research Council of the School of Rehabilitation, Tehran University of Medical Sciences (TUMS) (#36621).

### 2.2. Translation

The guidelines for the previously used forward-backward translation were followed by the expert panel [34,35,36]. First, two translators independently translated the English BESTest to Persian (forward translation). Then an expert panel (3 physiotherapists, an experienced methodologist, and 2 translators) reviewed the two versions and synthesized one Persian version for back translation. Two different English translators independently back translated the synthesized version to English. The expert panel with all the translators reviewed the documents and approved the pre-final Persian version to be sent to the developer (Prof. Fay B. Horak) for final approval. After approval of the Persian BESTtest by Prof. Horak, the final Persian BESTest emerged (https://www.bestest.us/files/3714/2472/0733/Persian_BESTest.pdf, accessed on 1 November 2023).

### 2.3. Participants

Participants were stroke patients referred from universities’ neurological and physiotherapy clinics. Inclusion criteria were: (1) stroke diagnosis, (2) hemiplegia resulting from stroke with a stable medical condition, (3) aged between 18 and 70 years, (4) ability to follow instructions, and (5) ability to stand and walk 6 m independently. The stroke diagnosis was made by neurologists based on the clinical and radiological findings.

Exclusion criteria were: (1) not willing to participate in the study, (2) inability to complete the tasks, (3) presence of balance disorders due to a medical condition other than stroke (e.g., Parkinson’s disease, vestibular disorders, untreated visual or hearing disabilities, pain, or impairments in the musculoskeletal system), (4) history of pathological vertigo, and (5) using medications affecting balance.

### 2.4. Test Procedure

The study was conducted in the neurological physiotherapy clinic of the school of rehabilitation of TUMS. At the first session, the participants’ demographic characteristics, including age, gender, and the time elapsed since the stroke, were collected. The study aims were explained to the participants, and written informed consent was obtained from them. In the next step, an experienced physiotherapist who was trained in the use of the BESTest, and had practiced performing the test items, assessed the participants’ balance. Every session included environmental preparation and it took approximately 35 min to complete the test. Both the test and retest were performed in the same environment for every patient. Patients were allowed to rest if they requested. The same physiotherapist assessed the patients again after one week.

### 2.5. Outcome Measure

The BESTest consists of 27 tasks and 36 items that evaluate six systems contributing to balance. Each item is scored from 0 (worst performance) to 3 (best performance). The sections’ scores were the sum of all the related items’ scores, and higher scores indicate better performance. The systems of the BESTest include biomechanical constraints (maximum possible score of 15), stability limits/verticality (maximum possible score of 21), transition/anticipatory postural adjustment (maximum possible score of 18), reactive postural responses (maximum possible score of 18), sensory orientation (maximum possible score of 15), and stability in gait (maximum possible score of 21). The total score of 108 is the sum of all the scores of all the sections.

### 2.6. Statistical Analysis

The mean and standard deviation (SD) was computed for continuous variables. Numbers and percentages were used for categorical variables. Intraclass correlation (ICC), a two-way random effect model, was used to determine the intra-rater reliability. An ICC value of 0.70 was considered as acceptable reliability, and scores were interpreted as excellent (>75), good (0.60–0.75), and fair (0.40–0.59) [37].

The standard error measurement (SEM, σ√1-ICC) and the smallest detectable change (SDC, 1.96 × SEM × √2) were calculated for the BESTest total score. The percentage of patients that scored the lowest or the highest possible score on the Persian BESTest were calculated for the presence of floor and ceiling effects (cut-off value ≥ 15%) [38]. Statistical analyses were performed using the SPSS version 17. *p* < 0.05 was considered statistically significant.

## 3. Results

A total of 18 participants were included in the study comprising 15 men and 3 women. The mean age was 56.39 (SD = 9.01). The mean time since the stroke was 50.0 months (SD = 35.08). Sixteen patients had ischemic stroke and two patients had hemorrhagic stroke. Ten patients had right hemiplegia. Characteristics of the individuals who participated in the study are presented in Table 1.

There was no issue with translating and adapting the BESTest into Persian, and all items were translated without any difficulties. During pilot testing, the therapist reported the test items that were understandable and easy to apply during the assessment.

The mean of the BESTest total score in the test and retest were 83.66 (SD = 11.98) and 82 (SD = 13.23), respectively. There were no floor and ceiling effects observed for the Persian BESTest total score (no patient scored the minimum or maximum on the Persian BESTtest). The intra-rater reliability of the BESTest total scores was high (ICC = 0.88, 95% CI = 0.73–0.95), and ICCs for sections ranged from 0.55 (95% CI = 0.12–0.80) for stability limits/verticality to 0.89 (95% CI = 0.55–0.96) for stability in gait (Table 2). The SEM and SDC of the BESTest total score were 8.33 and 22.82, respectively (Table 3).

## 4. Discussion

Balance impairments can significantly impact post-stroke patients’ quality of life [39]. Utilizing a reliable and valid instrument for balance evaluation to guide rehabilitation programs is essential. In this study, we translated and culturally adapted the BESTest into the Persian language and investigated its intra-rater reliability for the balance evaluation of Iranian post-stroke patients. The results showed that the Persian BESTest has excellent intra-rater reliability for balance evaluation in stroke patients and therefore can be used as a reliable tool for the assessment of balance in patients with stroke.

In the current study, the Persian version of the BESTest was developed and cross-culturally adapted for Persian patients with stroke. The successful development of the Persian BESTest indicates that the face and content validity of it is consistent with the original English BESTest and translated versions [25,29,31].

All patients in the study participated and completed the test procedure. There were no unexpected events or injuries that occurred during the testing with balance performances. As well, the rater reported no difficulties in conducting the assessment using the Persian BESTest. None of the patients had changes in their balance between the two test sessions. These indicate that the Persian BESTest was acceptable and feasible. The acceptability of the Persian BESTest is in line with those found for the translated versions of the BESTest [29,31,40,41].

Floor or ceiling effects were not present for the Persian BESTest total score. The lack of floor or ceiling effects indicates the content validity and responsiveness of the Persian BESTest. When there are no floor and ceiling effects for an instrument, patients with the lowest or highest possible score can be detected after an intervention. However, the responsiveness of the Persian BESTest was not evaluated in the current study which warrants an investigation designed in the context of a clinical trial. The lack of floor and ceiling effects observed in the present study is consistent with those reported for the translated versions of the BETSTest [31]. The floor and ceiling effects for the BESTest scores are not reported for the original version [25].

The Persian BESTest showed excellent intra-rater reliability. These findings are similar to those of previous studies [25,29,31,42] The original English version of the BESTest has been shown to have high inter- and intra-rater reliability (Horak et al., 2009). The test–retest reliability for the BESTest was high (ICC = 0.88) [42]. A study by Chinsongkram et al. in subacute stroke patients found excellent intra-rater reliability for the BESTest and its sections [40]. Rodrigues et al. (2014) evaluated the intra-rater reliability of the Brazilian BESTest in a sample of 16 chronic stroke patients and found the reliability for the total score (ICC = 0.98) and its sections (ICCs from 0.85 to 0.96) were excellent [40]. The findings indicate that the Persian BESTest in line with the original and other versions [25,31,40,41] can be used as a reliable tool for assessing the balance of patients with stroke.

The ICC for the stability limits/verticality section was the lowest in our study (0.55), in contrast to stability in the gait section, which was the highest (0.89). In the Rodrigues et al. study, ICC was the highest and lowest in the stability in gait and the reactive postural responses sections, respectively [41]. Chinsongkram reported the ICCs for sections ranging from 0.95 and 0.99 [40]. The reasons for discrepancies might be due to the differences in methodology. In the present study, one rater participated in the evaluation of intra-rater reliability and did not score the performances from the video as was done in the previous studies [40]. Future studies using more raters for the evaluation of intra-rater reliability may clarify the intra-rater reliability of the sections, particularly of stability limits/verticality in the Persian BESTest. Nevertheless, we should expect patients to perform differently in the BESTest sections, poor in some sections compared with other ones [25], thus affecting the perception of raters in the level of test performances.

The absolute reliability, presented by SEM and SDC, is an important reliability measure for clinical purposes. The SEM is used to determine the change in test scores which is a real beyond measurement error. The SEM value found in this study was 8.33. A previous study reported the SEM of 3.9/4.3 for two raters [31]. Therefore, the SEM value in our study indicates that the Persian BESTest is a useful tool to identify real changes in patients with stroke. However, the SDC is more important clinically than the SEM as it helps to identify real changes in patients. The SDC was calculated to determine whether an individual patient has achieved a real change after therapy [22]. We found that the SDC value of the Persian BESTest was 22.82. Previous studies reported the SDC as starting from 6.9 [31,43]. Hence, a change of more than 22.82 points in the Persian BESTest score must be observed after an intervention to be interpreted as real and clinically relevant.

There are several limitations of this study worth mentioning. First, in this study, we only evaluated patients with stroke, and our finding might not be applicable to other conditions affecting balance. Therefore, future studies are needed to evaluate the reliability of the Persian BESTest for balance evaluation in other patient groups with balance impairments. Second, we only evaluated intra-rater reliability, SDC, and SEM, and we did not assess other reliability and validity indexes. Future studies are needed to evaluate these indexes, such as construct validity, inter-rater reliability, responsiveness, and discriminative validity. Third, our sample size for reliability evaluation was suboptimal as we only included 18 patients. However, a study conducting power analyses indicated that a minimal sample of 10 participants provides 80% power to detect what would be considered an ICC of 0.70 [44].

## 5. Conclusions

In this study, we developed the Persian version of the BESTest and found it to be a reliable measure for the balance evaluation of Persian-speaking patients with stroke. This study suggests that the Persian version of the BESTest is a reliable tool which therapists can use for balance evaluation of patients with stroke and determine the system responsible for balance deficits. Further studies on the other psychometric characteristics of the Persian BESTest such as inter-rater reliability in larger sample sizes are warranted.

## Figures and Tables

**Table 1:** Basic and demographic characteristics of the participants.

Patients Number	Age (Year)	Duration since Stroke (Month)	Gender	Cause	Affected Side
1	65	24	Male	Ischemic	Right
2	56	72	Male	Ischemic	Left
3	66	47	Male	Ischemic	Left
4	55	136	Male	Hemorrhagic	Right
5	60	50	Male	Ischemic	Right
6	75	3	Male	Ischemic	Left
7	63	54	Male	Ischemic	Right
8	62	132	Female	Hemorrhagic	Left
9	46	50	Female	Ischemic	Right
10	61	36	Female	Ischemic	Left
11	43	60	Male	Ischemic	Left
12	46	8	Male	Ischemic	Right
13	45	37	Male	Ischemic	Left
14	57	36	Male	Ischemic	Right
15	45	42	Male	Ischemic	Right
16	63	35	Male	Ischemic	Right
17	49	54	Male	Ischemic	Right
18	58	24	Male	Ischemic	Left

**Table 2:** Sections and total scores in both sessions and intra-rater reliability for the BESTest.

Subscale	Session 1			Session 2			ICC (95% CI)	*p*-Value
	Min	Max	Mean (SD)	Min	Max	Mean (SD)		
Biomechanical constraints	6	14	11.61 (2.17)	6	14	10.61 (2.32)	0.72 (0.28–0.89)	**<0.001**
Stability limits/verticality	12	17	15.11 (1.27)	12	18	15.33 (1.45)	0.55 (0.12–0.80)	**0.008**
Transition/anticipatory postural adjustment	7	16	12.55 (2.38)	7	16	12.38 (2.50)	0.79 (0.53–0.91)	**<0.001**
Reactive postural responses	0	18	10.88 (5.01)	0	18	11.72 (5.16)	0.77 (0.49–0.90)	**<0.001**
Sensory orientation	12	15	14.61 (0.77)	12	15	14.66 (0.76)	0.76 (0.47–0.90)	**<0.001**
Stability in gait	10	21	18.83 (2.79)	9	21	18 (2.72)	0.89 (0.55–0.96)	**<0.001**
Total score	47 (43%)	101 (93%)	83.66 (11.98)	46 (42%)	102 (94%)	82 (13.23)	0.88 (0.73–0.95)	**<0.001**

**Table 3:** The standard error of measurement (SEM) and smallest detectable change (SDC) for the Persian BESTest.

Systems	SEM	SDC
Biomechanical constraints	2.24	6.06
Stability limits/verticality	1.6	4.38
Anticipatory responses	2.07	5.67
Postural responses	4.46	12.22
Sensory orientation	0.69	1.89
Stability in gait	1.78	4.87
Total score	8.33	22.82

## Data Availability

The data that support the findings of this study are available from the corresponding author, S.N., upon reasonable request. The data are not publicly available due to privacy and ethical restrictions.

## References

[1] Sarkar S., Chakraborty D., Bhowmik A., Ghosh M.K. (2019). Cerebral ischemic stroke: Cellular fate and therapeutic opportunities. Front. Biosci. -Landmark.

[2] Avan A., Digaleh H., Di Napoli M., Stranges S., Behrouz R., Shojaeianbabaei G., Amiri A., Tabrizi R., Mokhber N., Spence J.D. (2019). Socioeconomic status and stroke incidence, prevalence, mortality, and worldwide burden: An ecological analysis from the Global Burden of Disease Study 2017. BMC Med..

[3] Feigin V.L., Brainin M., Norrving B., Martins S., Sacco R.L., Hacke W., Fisher M., Pandian J., Lindsay P. (2022). World Stroke Organization (WSO): Global Stroke Fact Sheet 2022. Int. J. Stroke.

[4] Jaberinezhad M., Farhoudi M., Nejadghaderi S.A., Alizadeh M., Sullman M.J.M., Carson-Chahhoud K., Collins G.S., Safiri S. (2022). The burden of stroke and its attributable risk factors in the Middle East and North Africa region, 1990–2019. Sci. Rep..

[5] Sarrafzadegan N., Mohammadifard N. (2019). Cardiovascular disease in Iran in the last 40 years: Prevalence, mortality, morbidity, challenges and strategies for cardiovascular prevention. Arch. Iran. Med..

[6] Hosseini A.A., Sobhani-Rad D., Ghandehari K., Benamer H.T.S. (2010). Frequency and clinical patterns of stroke in Iran-Systematic and critical review. BMC Neurol..

[7] Mukherjee D., Levin R.L., Heller W. (2006). The cognitive, emotional, and social sequelae of stroke: Psychological and ethical concerns in post-stroke adaptation. Top. Stroke Rehabil..

[8] Lendraitienė E., Tamošauskaitė A., Petruševičienė D., Savickas R. (2017). Balance evaluation techniques and physical therapy in post-stroke patients: A literature review. Neurol. Neurochir. Pol..

[9] Zorowitz R.D., Gross E., Polinski D.M. (2002). The stroke survivor. Disabil. Rehabil..

[10] Jalayondeja C., Sullivan P.E., Pichaiyongwongdee S. (2014). Six-month prospective study of fall risk factors identification in patients post-stroke. Geriatr. Gerontol. Int..

[11] Hawkins K., Musich S., Ozminkowski R.J., Bai M., Migliori R.J., Yeh C.S. (2011). The burden of falling on the quality of life of adults with medicare supplement insurance. J. Gerontol. Nurs..

[12] Novitzke J.M. (2008). The rising cost of falling: Strategies to combat a common post-stroke foe. J. Vasc. Interv. Neurol..

[13] Iatridou G., Pelidou H.S., Varvarousis D., Stergiou A., Beris A., Givissis P., Ploumis A. (2017). The effectiveness of hydrokinesiotherapy on postural balance of hemiplegic patients after stroke: A systematic review and meta-analysis. Clin. Rehabil..

[14] Dominguez-Romero J.G., Molina-Aroca A., Moral-Munoz J.A., Luque-Moreno C., Lucena-Anton D. (2020). Effectiveness of Mechanical Horse-Riding Simulators on Postural Balance in Neurological Rehabilitation: Systematic Review and Meta-Analysis. Int. J. Environ. Res. Public Health.

[15] Chen L., Lo W.L., Mao Y.R., Ding M.H., Lin Q., Li H., Zhao J.L., Xu Z.Q., Bian R.H., Huang D.F. (2016). Effect of Virtual Reality on Postural and Balance Control in Patients with Stroke: A Systematic Literature Review. Biomed. Res. Int..

[16] Felius R.A.W., Geerars M., Bruijn S.M., Wouda N.C., Van Dieën J.H., Punt M. (2022). Reliability of IMU-based balance assessment in clinical stroke rehabilitation. Gait Posture.

[17] Pollock C.L., Eng J.J., Garland S.J. (2011). Clinical measurement of walking balance in people post stroke: A systematic review. Clin. Rehabil..

[18] Mancini M., Horak F.B. (2010). The relevance of clinical balance assessment tools to differentiate balance deficits. Eur. J. Phys. Rehabil. Med..

[19] Blum L., Korner-Bitensky N. (2008). Usefulness of the Berg Balance Scale in Stroke Rehabilitation: A Systematic Review. Phys. Ther..

[20] Naghdi S., Nakhostin Ansari N., Forogh B., Khalifeloo M., Honarpisheh R., Nakhostin-Ansari A. (2020). Reliability and Validity of the Persian Version of the Mini-Balance Evaluation Systems Test in Patients with Stroke. Neurol. Ther..

[21] Chinsongkram B., Chaikeeree N., Saengsirisuwan V., Horak F.B., Boonsinsukh R. (2016). Responsiveness of the Balance Evaluation Systems Test (BESTest) in people with subacute stroke. Phys. Ther..

[22] Oyama C., Otaka Y., Onitsuka K., Takagi H., Tan E., Otaka E. (2018). Reliability and validity of the Japanese version of the mini-balance evaluation systems test in patients with subacute stroke. Prog. Rehabil. Med..

[23] Whitney S., Wrisley D., Furman J. (2003). Concurrent validity of the Berg Balance Scale and the Dynamic Gait Index in people with vestibular dysfunction. Physiother. Res. Int..

[24] Joshua A.M., Karnad S.D., Nayak A., Suresh B.v., Mithra P., Unnikrishnan B. (2017). Effect of foot placements during sit to stand transition on timed up and go test in stroke subjects: A cross sectional study. NeuroRehabilitation.

[25] Horak F.B., Wrisley D.M., Frank J. (2009). The balance evaluation systems test (BESTest) to differentiate balance deficits. Phys. Ther..

[26] Priplata A.A., Patritti B.L., Niemi J.B., Hughes R., Gravelle D.C., Lipsitz L.A., Veves A., Stein J., Bonato P., Collins J.J. (2006). Noise-enhanced balance control in patients with diabetes and patients with stroke. Ann. Neurol..

[27] Lee N.G., You J.S., Chung H.Y., Jeon H.S., Choi B.S., Lee D.R., Park J.M., Lee T.H., Ryu I.T., Yoon H.S. (2018). Best Core Stabilization for Anticipatory Postural Adjustment and Falls in Hemiparetic Stroke. Arch. Phys. Med. Rehabil..

[28] De Oliveira C.B., De Medeiros Í.R.T., Frota N.A.F., Greters M.E., Conforto A.B. (2008). Balance control in hemiparetic stroke patients: Main tools for evaluation. J. Rehabil. Res. Dev..

[29] Haselwander M., Henes Y., Weisbrod M., Diermayr G. (2023). Balance Evaluation Systems Test: German translation, cultural adaptation and preliminary results on psychometric properties. Z. Gerontol. Geriatr..

[30] Dominguez-Olivan P., Gasch-Gallen A., Aguas-Garcia E., Bengoetxea A. (2020). Validity and reliability testing of the Spanish version of the BESTest and mini-BESTest in healthy community-dwelling elderly. BMC Geriatr..

[31] Hamre C., Botolfsen P., Tangen G.G., Helbostad J.L. (2017). Interrater and test-retest reliability and validity of the Norwegian version of the BESTest and mini-BESTest in people with increased risk of falling. BMC Geriatr..

[32] Torres-Narvaez M.R., Luna-Corrales G.A., Rangel-Pineros M.C., Pardo-Oviedo J.M., Alvarado-Quintero H. (2018). Transcultural adaptation to the Spanish language of the Balance Evaluation Systems Test (BESTest) in older adults. Rev. Neurol..

[33] Jeon Y.J., Kim G.M. (2018). A Study of Translation Conformity on Korean Version of a Balance Evaluation Systems Test. Phys. Ther. Korea.

[34] Beaton D.E., Bombardier C., Guillemin F., Ferraz M.B. (2000). Guidelines for the process of cross-cultural adaptation of self-report measures. Spine.

[35] Naghdi S., Ansari N.N., Raji P., Shamili A., Amini M., Hasson S. (2016). Cross-cultural validation of the Persian version of the Functional Independence Measure for patients with stroke. Disabil. Rehabil..

[36] Nakhostin Ansari N., Naghdi S., Eskandari Z., Salsabili N., Kordi R., Hasson S. (2016). Reliability and validity of the Persian adaptation of the Core Outcome Measure Index in patients with chronic low back pain. J. Orthop. Sci..

[37] Fleiss J.L., Levin B., Cho Paik M. (2004). Statistical Methods for Rates and Proportions.

[38] Terwee C.B., Van Der Windt D.A., Dekker J., Bot S.D.M., De Boer M.R., Bouter L.M., Knol D.L., de Vet H.C.W. (2007). Quality Criteria Were Proposed for Measurement Properties of Health Status Questionnaires.

[39] Schmid A.A., van Puymbroeck M., Altenburger P.A., Miller K.K., Combs S.A., Page S.J. (2013). Balance is associated with quality of life in chronic stroke. Top. Stroke Rehabil..

[40] Chinsongkram B., Chaikeeree N., Saengsirisuwan V., Viriyatharakij N., Horak F.B., Boonsinsukh R. (2014). Reliability and Validity of the Balance Evaluation Systems Test (BESTest) in People With Subacute Stroke. Phys. Ther..

[41] Rodrigues L.C., Marques A.P., Barros P.B., Michaelsen S.M. (2014). Reliability of the Balance Evaluation Systems Test (BESTest) and BESTest sections for adults with hemiparesis. Braz. J. Phys. Ther..

[42] Leddy A.L., Crowner B.E., Earhart G.M. (2011). Functional Gait Assessment and Balance Evaluation System Test: Reliability, Validity, Sensitivity, and Specificity for Identifying Individuals With Parkinson Disease Who Fall. Phys. Ther..

[43] Huang M.H., Miller K., Smith K., Fredrickson K., Shilling T. (2016). Reliability, Validity, and Minimal Detectable Change of Balance Evaluation Systems Test and Its Short Versions in Older Cancer Survivors: A Pilot Study. J. Geriatr. Phys. Ther..

[44] Bujang M.A., Baharum N. (2017). A simplified guide to determination of sample size requirements for estimating the value of intraclass correlation coefficient: A review. Arch. Orofac. Sci. J. Sch. Dent. Sci. USM Arch Orofac Sci..

